# Early Antiretroviral Therapy reduces the incidence of otorrhea in a randomized study of early and deferred antiretroviral therapy: Evidence from the Children with HIV Early Antiretroviral Therapy (CHER) Study

**DOI:** 10.1186/1756-0500-4-448

**Published:** 2011-10-26

**Authors:** Clotilde Hainline, Reghana Taliep, Gill Sorour, Sharon Nachman, Helena Rabie, Els Dobbels, Anita Janse van Rensburg, Morna Cornell, Avy Violari, Shabir A Madhi, Mark F Cotton

**Affiliations:** 1 School of Medicine, Stony Brook University, Stony Brook, New York, USA; 2 Division of Infectious Diseases, Stony Brook University, Stony Brook, New York, USA; 3 Department of Paediatrics and Child Health and Children's Infectious Diseases Clinical Research Unit (KID-CRU), Tygerberg Children's Hospital and Faculty of Health Sciences, Stellenbosch University, Tygerberg, South Africa; 4 School of Public Health, University of Cape Town, South Africa; 5 Perinatal HIV Research Unit, University of Witwatersrand, South Africa; 6 Department of Science and Technology/National Research Foundation: Vaccine Preventable Diseases, University of the Witwatersrand, South Africa

## Abstract

**Background:**

Although otorrhea occurs commonly in HIV-infected infants, there are few data. We compared the incidence of otorrhea in infants receiving early vs deferred ART in the Children with HIV Early Antiretroviral (CHER) trial. Infants aged 6 to 12 weeks of age with confirmed HIV infection and a CD4 percentage greater than or equal to 25% were randomized to early or deferred ART at two sites in South Africa. Medical records from one study site were reviewed for otorrhea.

**Findings:**

Data were reviewed from the start of the trial in July 2005 until 20 June 2007, when the Data Safety Monitoring Board recommended that randomization to the deferred arm should stop and that all infants in this arm be reviewed for commencing antiretroviral therapy. Infants entered the study at a median of 7.4 weeks of age. Eleven of 38 (29%) on deferred therapy and 7 of 75 (9%) in the early-therapy group developed otorrhea (risk ratio 3.1, 95% confidence interval (CI) 1.31-7.36; p = 0.01).

**Conclusions:**

Early initiation of antiretroviral therapy is associated with significantly less otorrhea than when a deferred strategy is followed.

**Trial registration:**

NCT00102960. ClinicalTrials.Gov

## Background

Chronic suppurative otitis media (CSOM), usually accompanied by otorrhea, is a major cause of hearing impairment in children, especially in developing countries. Management is either unsatisfactory or very difficult [1]. Otorrhea typically occurs after perforation of the tympanic membrane as a complication of acute otitis media, with subsequent discharge of pus into the external ear canal. Acute otitis media is hard to diagnose in young infants due to non-specificity of presenting symptoms and the difficulty of visualizing the tympanic membrane [2]. Otorrhea, however, is easy to visualize and does not require any equipment [1].

The study was a retrospective folder search for otorrhea identified in the clinical notes and assumed due to a perforation of the tympanum, which is the most common cause. External otitis media was excluded clinically. Otitis media and otorrhea are World Health Organization (WHO) stage II events for HIV in children [3]. Both occur frequently in HIV-infected children [4-6]. Antiretroviral therapy (ART) was associated with a reduction in otitis media and CSOM in a retrospective cohort [7].

In a sub-analysis of a prospective randomized strategy trial, we compared otorrhea in young HIV-infected infants receiving early versus delayed ART. Infants aged 6 to 12 weeks of age with confirmed HIV infection and a CD4 percentage greater than or equal to 25% were randomized to deferred ART according to South African guidelines [8] (the deferred-therapy group) or immediate ART until 1 or 2 years of age (the early-therapy groups), followed by treatment interruption.

## Methods

### Background

The Children with HIV Early Antiretroviral Therapy (CHER) trial is a prospective randomized study evaluating ART strategies in two centers, the Children's Infectious Diseases Clinical Research Unit (KID-CRU), Tygerberg, Cape Town and the Perinatal HIV Research Unit, Soweto, South Africa. All infants were confirmed HIV-positive by polymerase chain reaction (PCR) test. Immunologic criteria for initiating ART in the deferred-therapy group were a CD4 percentage below 20% or, for children younger than 12 months, a CD4 percentage below 25% or a CD4 count below 1000 cells per mm^3^, according to WHO guidelines updated in 2006 [9]. ART consisted of lopinavir/ritonavir, zidovudine and lamivudine and commenced at baseline in the early ART group. The primary outcomes of interest were death and disease progression.

After randomization, infants were seen every 4 weeks until week 24, every 8 weeks until week 48 and at 12 weekly intervals thereafter. Infants were staged according to criteria of the Centers for Diseases Control and Prevention with stage C and predefined severe stage B criteria used for the study end point of disease progression [10]. These criteria approximated WHO stage 3 and 4 [3]. Persistent otorrhea (duration not defined) is a WHO stage 2 event.

The majority of infants were co-enrolled in a companion study of the 7-valent conjugated Prevnar^® ^*Streptococcus pneumoniae *vaccine [11]: 36/38 (95%) in the deferred arm and 65/75 (87%) in the early treatment arm. All infants also received the conjugated *Haemophilus influenzae *B vaccine according to the national immunization program and trimethophrim-sulfamethoxazole (TMP-SMX) prophylaxis.

The CHER study commenced in July 2005. On June 20^th^, 2007, the Data Safety Monitoring Board (DSMB) stopped the deferred arm after a median of 40 (interquartile range 24 to 58) weeks on study when initial data showed that early ART initiation reduced HIV-related disease progression and mortality by 75% [12].

### Study procedures

Data for the present report were from KID-CRU only. The charts of all infants with CD4% greater than or equal to 25% enrolled in CHER at KID-CRU from August 2005 through June 20, 2007 were reviewed.

Otorrhea was classified as mild (a single episode lasting less than 1 month) or severe (either a single episode lasting more than 1 month or more than 1 episode over the study period). This classification is a modification of that proposed by Sabella [13]. Although WHO accepts more than two weeks of otorrhea for a diagnosis of CSOM, many otorrhinolaryngologists require a duration longer than 3 months [1]. The Committee for Clinical Trials at Stellenbosch University approved both the CHER study and the present sub-analysis (Reference number: M04/07/033A). The trial was conducted in accordance with the Helsinki declaration. A parent, usually the mother, or legal guardian gave written informed consent for each child's participation.

### Statistical Analysis

All statistical analyses, including the calculation of risk ratios and confidence intervals (CI), were performed using STATA version 11.0 (STATA Corporation, College Station, TX). Chi-square and Fisher exact tests were performed for categorical variables and Wilcoxon rank tests were performed for continuous variables. All tests were 2-tailed. Significance level was set at 0.05.

## Results

Between August 2005 and February 2007, 113 infants were enrolled. Thirty-eight were assigned to deferred therapy and 75 to early therapy. Baseline characteristics were well matched apart from infants on early therapy being slightly older (7.7 vs 7.1 weeks) and a trend to better weight-for-age-Z score in the deferred group (-0.16 vs -0.55) (Table 1). The CD4 percentages and counts were high: CD4% was 35.2% in the early ART group and 35.8% in the deferred groups, and absolute CD4 count 1835 vs 1728 cells/mm^3 ^respectively. Two infants randomized to receive early ART had episodes of otorrhea before baseline and were excluded from the analysis of otorrhea events. Median (inter-quartile range) follow-up time was 9.6 (7.2-12.8) months. Median (IQR) time on ART was 9.0 (5.5-9.3) months in the early arm and 3.5 (2.3-5.3) months in the deferred group. Six participants in the deferred group had otorrhea on ART. Among these, median time on ART before otorrhea was 4.1 (0.9-4.6) months. Overall, median CD4 count increased from 1255 cells per mm^3 ^pre-otorrhea to 1352 afterwards: in the deferred group, CD4 count decreased from 1286 to 1160; in the early group, CD4 count increased from 1213 to 1774 cells per mm^3^.

**Table 1:** Baseline characteristics of infants receiving early vs deferred ART

Variable	Early ART (n = 75)	Deferred ART (n = 38)	Total (n = 113)	P-value
Female (%)	44 (59%)	23 (61%)	67 (59%)	1
Median age (weeks)	7.7	7.0	7.4	0.004
	(7.1 to 9.4)	(6.5 to 8.5)	(6.9 to 9.2)	
Median weight for age Z-score	-0.55	-0.16	-0.47	0.08
	(-1.35 to 0.29)	(-1.02 to 0.55)	(-1.24 to 0.33)	
WHO Stage				
I	23 (31%)	14 (37%)	37 (33%)	0.67
II	14 (19%)	5 (13%)	19 (17%)	
III	38 (50%)	19 (50%)	57 (50%)	
Median CD4 %	35.2	35.8	35.6	0.89
	(28.4 to 40.6)	(30.0 to 41.8)	(28.9 to 40.6)	
Median CD4 count (cells/mm^3^)	1835	1728	1777	0.27
	(1362 to 2645)	(1174 to 2195)	(1325 to 2509)	

Continuous data (IQR)

Eighteen infants developed at least one episode of otorrhea after baseline: 11 of 38 (29%) in the deferred-therapy group and 7 of 75 (9%) in the early-therapy groups (risk ratio 3.10, 95% CI, 1.31-7.36; p = 0.01). Evaluation for severe otorrhea showed a similar trend, but did not reach statistical significance. Seven (18.4%) in the deferred arm and 6 (8%) in the early ART arm had severe disease (risk ratio 1.74, 95% CI, 0.97-3.11; p = 0.10).

Three infants, one in the deferred-therapy group and two in the early-therapy groups, each had 2 separate events of otorrhea. In the deferred-therapy group, 40% of infants had not initiated ART at onset of otorrhea.

Eight infants had otorrhea fluid submitted for culture, 4 from the deferred and 4 on early ART. Two *S. pneumonia *isolates were from infants on the deferred arm. Of two *Staphylococcus aureus *isolates, one was methicillin sensitive and one was resistant. The other isolates included Pseudomonas, Proteus and Serrattia species and one of *Kelbsiella pneumonia*. Five of 7 organisms were resistant to TMP-SMX.

## Discussion

In this prospective randomized study, we showed that early initiation of ART provided significant protection against otorrhea compared to deferred ART, initiated on the basis of either CD4 depletion or progression of HIV disease. Infants in the deferred arm had 3 times the risk of developing otorrhea compared with those who started treatment immediately. Nevertheless, even in the early therapy group, the incidence was relatively high (9%). In a cross-sectional survey from Swaziland before the advent of HIV, the prevalence of otorrhoea in children attending grade 1 was only 1.3% [14].

Of note is that all infants entered the study being relatively asymptomatic and with high baseline CD4 percentages at a median of 7.4 weeks of age. We documented a high incidence of otorrhea in the first year of life. Our study provides additional evidence of the benefits of ART for infants. Preventing otorrhea in HIV-infected infants improves their quality of life, preserves hearing and reduces the burden on the health system. Interestingly, the CD4 counts were relatively high in both groups, likely due to careful monitoring and ready access to ART in a trial setting. We infer that ART must have a qualitative benefit on CD4 functioning.

Our results are similar to those from a retrospective cohort study from Brazil, which found that ART comprising three antiretrovirals was associated with a significantly lower prevalence of otorrhea (7.1%) than children receiving only one or two antiretrovirals (20.8%) (p = 0.02) [7]. The mean age in this study was 6.6 (± 2.5) years, far older than in our study. We now confirm that their observations are already noted in the first year of life. In an earlier study from our unit, the prevalence of otorrhea before the widespread availability of ART was 32%, close to the prevalence in our deferred-therapy group, and was linked to CD4 depletion [5]. Had we included infants with a CD4 percentage below 25% in CHER, it is likely that we would have documented a higher prevalence of otorrhea.

The majority of events in the delayed group occurred on therapy. Possible explanations may include immune reconstitution inflammatory syndrome as well as better qualitative antibody production with early antiretroviral therapy [11].

Our study is strengthened by the fact that it was a prospective randomized trial. In addition, the study population represents the broader infant population accessing ART through the public health system, making the results widely generalisable.

There are a number of limitations to our study. Infants were not evaluated by an otorrhinolaryngologist. Perforation of the tympanic membrane was not uniformly documented. Also, we did not consistently submit otorrhea specimens for bacterial culture or serotyping of *S. pneumonia *isolates. We were therefore unable to evaluate the effect of immunization with the conjugated pneumococcal vaccine. We have, however, demonstrated in the companion study to CHER that early initiation of ART is associated with improved qualitative function of specific antibodies to serotypes in vaccine [11]. An intriguing finding in the present study was that the *S. pneumonia *isolates were both from infants where ART was deferred.

## Conclusion

Early ART in young HIV-infected infants significantly reduces the incidence of otorrhea, a condition associated with much morbidity.

## Abbreviations

ART: antiretroviral therapy; CHER: Children with HIV Early Antiretroviral (CHER) trial; CI: confidence interval; CIPRA-SA: Comprehensive International Programme of Research on AIDS - South Africa; CSOM: chronic suppurative otitis media; DSMB: Data Safety Monitoring Board; KID-CRU: Children's Infectious Diseases Clinical Research Unit; WHO: World Health Organization

## Competing interests

The authors declare that they have no competing interests.

## Authors' contributions

CH undertook the review of medical records and wrote the first version of the manuscript. RT checked the data and contributed to the manuscript. GS assisted with medical record review and Ethics Committee submission, supervised CH (a pre-med student) and contributed to the manuscript. SN mentored CH and reviewed the manuscript. HR provided clinical leadership and reviewed the manuscript. ED assisted with identification of subjects and review of the manuscript. AJvR assisted with access to the medical records and reviewed the manuscript. MC and MFC did statistical analysis. MC reviewed the manuscript. AV (CHER co-PI) and SAM reviewed the manuscript. MFC was the overall leader of the sub-analysis who conceived of the study and took overall responsibility for the manuscript during all phases. All authors read and approved the final manuscript.

## References

[B1] World Health Organization Chronic suppurative otitis media. Burden of Illness and Management Options 2004 Geneva

[B2] Berkun Y Nir-Paz R Ami AB Klar A Deutsch E Hurvitz H Acute otitis media in the first two months of life: characteristics and diagnostic difficulties Arch Dis Child 2008 93 8 690 694 10.1136/adc.2007.127522 18337275

[B3] World Health Organization WHO case definitions of HIV for surveillance and revised clinical staging and immunological classification for HIV-related disease in adults and children 2006 Geneva: world Health Organization

[B4] Hoare S HIV infection in children?impact upon ENT doctors Int J Pediatr Otorhinolaryngol 2003 67 S85 S90 14662174 10.1016/j.ijporl.2003.08.036

[B5] Karpakis J Rabie H Howard J Janse van Rensburg A Cotton MF Otorrhoea is a marker for symptomatic disease in HIV-infected children S Afr Med J 2007 97 12 1292 1294 18264613

[B6] Shapiro NL Novelli V Otitis media in children with vertically-acquired HIV infection: the Great Ormond Street Hospital experience Int J Pediatr Otorhinolaryngol 1998 45 1 69 75 10.1016/S0165-5876(98)00089-5 9804022

[B7] Miziara ID Weber R Filho BCA Neto CDP Otitis media in Brazilian human immunodeficiency virus infected children undergoing antiretroviral therapy The Journal of Laryngology & Otology 2007 121 11 17319998 10.1017/S0022215107006093

[B8] National Department of Health Department of Health National Antiretroviral Treatment Guidelines 2004 1 Pinetown: Jacana Publishers

[B9] World Health Organization Antiretroviral therapy of HIV infection in infants and children in resource-limited settings: towards universal access. Recommendations for a public health approach 2006 Geneva 23741772

[B10] Centers For Disease Control and Prevention Revised classification system for human immunodeficiency virus infection in children less than 13 years of age Morb Mortal Wkly Rep 1994 43 1 10

[B11] Madhi S Adrian P Cotton MF McIntyre J Jean-Phillipe P Meadows S Nachman S Klugman KP Käyhty H Violari A Effect of HIV exposure and antiretroviral therapy on quantitative antibody concentration to pneumococcal conjugate vaccine (PCV) in infants5th IAS Conference on HIV Pathogenesis, Treatment andPrevention 2009 Cape Town

[B12] Violari A Cotton MF Gibb DM Babiker AG Steyn J Madhi SA Jean-Philippe P McIntyre JA Early antiretroviral therapy and mortality among HIV-infected infants N Engl J Med 2008 359 21 2233 2244 10.1056/NEJMoa0800971 19020325 PMC2950021

[B13] Sabella C Management of otorrhea in infants and children Pediatr Infect Dis J 2000 19 10 1007 1008 10.1097/00006454-200010000-00014 11055606

[B14] Swart SM Lemmer R Parbhoo JN Prescott CA A survey of ear and hearing disorders amongst a representative sample of grade 1 schoolchildren in Swaziland International Journal of Pediatric Otorhinolaryngology 1995 32 1 23 34 10.1016/0165-5876(94)01109-B 7607818

